# Per- and polyfluoroalkyl substances (PFAS) and cancer: knowledge mapping and emerging trends based on a bibliometric analysis

**DOI:** 10.3389/fonc.2026.1880116

**Published:** 2026-10-19

**Authors:** Siyin Liu, Guoqing Zhang, Meijing Wang, Jiaguo Huang

**Affiliations:** 1 Department of Breast and Thyroid Surgery, People’s Hospital of Xinjiang Uygur Autonomous Region, Urumgi, China; 2 Department of Clinical Nutrition, West China Hospital, Sichuan University, Chengdu, China; 3 College of Traditional Chinese Medicine, Xinjiang Medical University, Xinjiang, China; 4 Department of Urology, Affiliated Xiaoshan Hospital, Hangzhou Normal University, Hangzhou, China

**Keywords:** bibliometric analysis, cancer, Citespace, per- and polyfluoroalkyl substances, VOSviewer

## Abstract

**Objectives:**

To systematically map the global publication landscape, collaboration patterns, thematic structure, and emerging research activity in the literature on per- and polyfluoroalkyl substances (PFAS) and cancer.

**Methods:**

A systematic bibliometric analysis of the literature on PFAS and cancer published over the past 20 years was conducted using the Web of Science Core Collection and Scopus database and analyzed with CiteSpace and VOSviewer.

**Results:**

A bibliometric analysis of 1126 publications revealed the principal publication and thematic patterns in PFAS–cancer research. The field has grown substantially since 2022, with the United States and China leading the global research output. At the institutional level, the Chinese Academy of Sciences was the most productive institution, while *Environment International* is the most prolific journal. Keyword clustering showed a well-defined and internally consistent thematic structure, encompassing specific PFAS compounds, cancer types, endocrine-disrupting effects, exposure assessment, emerging contaminants, and computational toxicology. Temporal analysis indicated a broadening of research attention from legacy compounds, particularly PFOA and PFOS, toward the wider PFAS class and a more diverse range of cancer sites. Keyword burst detection and timeline analyses identified network toxicology, molecular docking, machine learning, and lipid metabolism as areas of rapidly increasing recent research activity.

**Conclusions:**

This study offers a comprehensive overview of the global research landscape concerning the association between PFAS and cancer.

## 1 Introduction

The escalating global burden of cancer has established it as a principal cause of death and disability, presenting a significant threat to public health and socioeconomic stability. The World Health Organization (WHO) reports a continuous rise in the global cancer burden, with approximately 20 million new cases and 9.7 million deaths recorded in 2022. Future projections suggest this burden will expand to 35 million new cases by 2050, considering global population growth and aging (1). The development of cancer is understood as a complex, multistage, and polygenic process, arising from the cumulative interplay of genetic susceptibility, environmental exposures, and lifestyle patterns over time. Its etiology stems from prolonged interactions between genetic predisposition, environmental exposures, and lifestyle factors. In addition to established risk factors like dietary imbalances, obesity, and radiation exposure, chronic exposure to environmental pollutants, notably persistent organic pollutants, is increasingly recognized as a critical external driver of tumor development (2).

Per- and polyfluoroalkyl substances (PFAS) are synthetic persistent organic pollutants used extensively in industrial and consumer applications since the 1940s (3). Their chemical stability has led to their ubiquitous presence and widespread global distribution in soil, water, and the atmosphere (4). Concentrations of key PFAS like perfluorooctanoic acid (PFOA) and perfluorooctane sulfonate (PFOS) now exceed regulatory safety limits in environmental media across many nations, signifying that PFAS pollution has breached planetary boundaries and evolved into a critical global public health threat (5). Humans are exposed to PFAS through their diet, air, and water, and as a result, these chemicals are found in the blood of the vast majority of the population (6, 7). The bioaccumulative nature of PFAS is pronounced, with serum elimination half-lives in the human body ranging from approximately 3 to 8 years (8). This characteristic of persistent internal exposure is linked to toxicity across multiple organ systems and an elevated risk for numerous chronic conditions, thus constituting a continuous and significant threat to human well-being.

Studies have linked PFAS to numerous health hazards, including developmental, reproductive, immune, liver, and neurotoxicity (9). In recent years, scientific inquiry has increasingly focused on the potential role of PFAS in the development of various systemic cancers, making the association between PFAS exposure and oncology a prominent research hotspot (10–12). In 2023, the WHO’s International Agency for Research on Cancer (IARC) classified PFOA as “carcinogenic to humans” (Group 1) and PFOS as “possibly carcinogenic to humans” (Group 2B), citing sufficient evidence in experimental animals and strong mechanistic evidence in humans (13).

Despite numerous studies investigating the association between PFAS and cancer, a comprehensive bibliometric analysis of its knowledge structure is currently absent. The discipline of bibliometrics involves a thorough investigation of scholarly output, focusing on indicators such as publication output, citation patterns, collaboration networks, and thematic relationships. Such analysis uncovers the fundamental components and intellectual architecture of a specific field, thereby enhancing comprehension of its contemporary state, research hotspots, and prospective developments (14). In light of the pervasive human exposure to PFAS and their carcinogenic potential, this study employs bibliometric analysis to map the research landscape of PFAS and cancer, aiming to provide guidance for future research and practice.

## 2 Methods

### 2.1 Data source and collection

The reporting of this bibliometric analysis conforms to the guidelines outlined in the Preliminary guideline for reporting bibliometric reviews of the biomedical literature (BIBLIO) checklist for biomedical literature (15). The Web of Science and Scopus database were utilized as the primary search platform for this study. They were selected due to its comprehensive coverage of high-impact journals and its ability to provide more comprehensive and reliable bibliometric analyses. We retrieved literature addressing the association between PFAS and cancer from the Web of Science Core Collection (WoSCC) and Scopus. The dataset was refined to include only articles and reviews published in English from January 1, 2006, through March 31, 2026. The search was conducted on April 1, 2026, with the following search strategy: (TS = (“polyfluoroalkyl substance*” OR “perfluoroalkyl substance*” OR (perfluoro* NEAR/3 acid*) OR “perfluorooctanoate*” OR “perfluoro* sulfonate*” OR “perfluorooctanesulfonate” OR “per(poly)fluoroalkyl substance*” OR “perfluorinated compound*” OR “perfluoroalkyl acid*” OR “perfluorocarboxylic acid*” OR “perfluoroalkyl carboxylic acid*” OR “perfluoroalkyl sulfonate*” OR “perfluorosulfonic acid*” OR “perfluorinated phosphonic acid*” OR “perfluoroalkyl phosphonic acid*” OR “perfluoroether carboxylic acid*” OR “perfluoroether sulfonic acid*” OR “perfluorooctane sulfonate” OR “perfluorooctane sulfonic acid” OR “perfluorooctanoic acid” OR “perfluorooctanoate”)) AND TS = (cancer* OR neoplas* OR oncolog* OR metasta* OR malignan* OR adenoma* OR tumor* OR tumour* OR adenocarcinoma* OR blastoma* OR carcinogen* OR carcinom* OR carcinosarcoma* OR chordoma* OR germinoma* OR gonadoblastoma* OR hepatoblastoma* OR hodgkin* OR leukemi* OR lymphangioma* OR lymphangiomyoma* OR lymphangiosarcoma* OR lymphom* OR melanom* OR meningioma* OR mesenchymoma* OR mesonephroma* OR neuroma* OR nsclc OR oncogen* OR paraneoplastic* OR plasmacytoma* OR precancerous* OR sarcoma* OR teratocarcinoma* OR teratoma* OR nephroblastoma* OR nefroblastoma* OR retinoblastoma* OR rhabdomyosarcoma* OR osteosarcoma* OR neuroblastoma* OR Kahler* OR myeloma* OR oncological) in WoSCC and TITLE-ABS-KEY((“polyfluoroalkyl substance*”) OR (“perfluoroalkyl substance*”) OR (perfluoro* W/3 acid*) OR (“perfluorooctanoate*”) OR (“perfluoro* sulfonate*”) OR (“perfluorooctanesulfonate”) OR (“per(poly)fluoroalkyl substance*”) OR (“perfluorinated compound*”) OR (“perfluoroalkyl acid*”) OR (“perfluorocarboxylic acid*”) OR (“perfluoroalkyl carboxylic acid*”) OR (“perfluoroalkyl sulfonate*”) OR (“perfluorosulfonic acid*”) OR (“perfluorinated phosphonic acid*”) OR (“perfluoroalkyl phosphonic acid*”) OR (“perfluoroether carboxylic acid*” OR “perfluoroether sulfonic acid*” OR “perfluorooctane sulfonate” OR “perfluorooctane sulfonic acid” OR “perfluorooctanoic acid” OR “perfluorooctanoate”)) AND TITLE-ABS-KEY(cancer* OR neoplas* OR oncolog* OR metasta* OR malignan* OR adenoma* OR tumor* OR tumour* OR adenocarcinoma* OR blastoma* OR carcinogen* OR carcinom* OR carcinosarcoma* OR chordoma* OR germinoma* OR gonadoblastoma* OR hepatoblastoma* OR hodgkin* OR leukemi* OR lymphangioma* OR lymphangiomyoma* OR lymphangiosarcoma* OR lymphom* OR melanom* OR meningioma* OR mesenchymoma* OR mesonephroma* OR neuroma* OR nsclc OR oncogen* OR paraneoplastic* OR plasmacytoma* OR precancerous* OR sarcoma* OR teratocarcinoma* OR teratoma* OR nephroblastoma* OR nefroblastoma* OR retinoblastoma* OR rhabdomyosarcoma* OR osteosarcoma* OR neuroblastoma* OR Kahler* OR myeloma* OR oncological) in Scopus. The literature screening process is illustrated in **Figure 1**.

**Figure 1:**
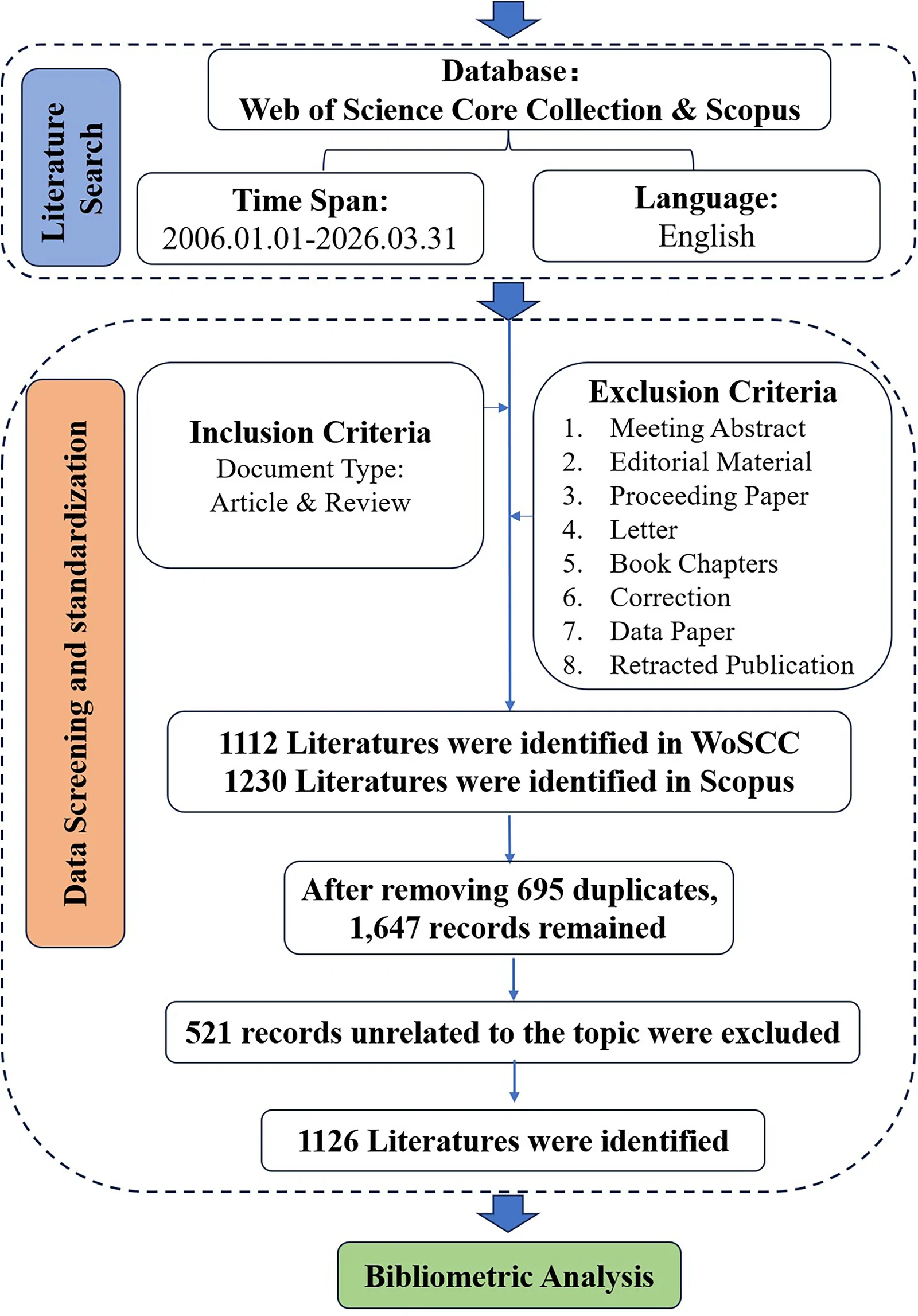
Flow diagram of study selection and data analysis.

### 2.2 Data preprocessing and deduplication

Records retrieved from the WoSCC and Scopus were exported separately as Microsoft Excel files with full bibliographic information and cited references. The records from the two databases were subsequently combined into a single Excel spreadsheet. Before bibliometric analysis, the merged dataset underwent duplicate removal and eligibility screening.

Duplicate records were identified primarily by comparing publication titles. Records with identical or highly similar titles were considered potential duplicates and were further verified by comparing the authors, journal title, and publication year. When the same publication was indexed in both WoSCC and Scopus, only one record was retained, while the duplicate entry was removed from the merged dataset. A total of 2,342 records were initially retrieved from the two databases. After 695 duplicate records were removed, 1,647 unique records remained for eligibility screening.

Two researchers (S.L. and G.Z.) independently screened the titles and abstracts of the remaining records. Full texts were further examined when the relevance of a publication could not be determined from its title and abstract. Records were included when PFAS exposure, occurrence, biomonitoring, toxicology, or risk assessment was substantively investigated in relation to cancer incidence, cancer risk, carcinogenicity, tumor-related biological processes, or explicitly cancer-relevant mechanisms. Records were excluded when PFAS or cancer-related terminology appeared only incidentally, when PFAS were not a substantive exposure or analyte, or when the publication did not contain content directly relevant to cancer. Disagreements were resolved through discussion; when consensus could not be reached, a third researcher (J.H.) made the final decision. After title, abstract, and, when necessary, full-text screening, 521 records unrelated to the research topic were excluded, leaving 1,126 publications in the final bibliometric dataset. Examples of excluded records and the reasons for exclusion are provided in **Supplementary Table S1**.

### 2.3 Data analysis and visualization

Data analysis was performed using a combination of statistical and visualization software. Microsoft Excel 2019 was used to compile descriptive statistics and chart annual publication output. To visualize the intellectual structure and evolution of the field, we utilized VOSviewer (1.6.20) and CiteSpace (6.4.R1) for network mapping and identifying research frontiers. This multi-tool approach enabled a comprehensive analysis of the literature’s authors, institutions, countries, journals, and keywords.

CiteSpace was used to construct the visual knowledge maps. The analysis covered the period from 2006 to 2026, with each time slice representing one year. Nodes were selected using the g-index with a scaling factor (k) of 25. For keyword co-occurrence analysis, cluster labels were generated using the log-likelihood ratio method. Keyword citation bursts were detected using the burst-detection function embedded in CiteSpace. The detection model was configured with two states, an α ratio of 2.0, a γ value of 0.1, and a minimum burst duration of one year. Keywords were ranked according to burst strength, and the 10 keywords with the strongest citation bursts were retained for visualization.

To reduce the influence of publication age on citation-based comparisons, normalized citation scores provided by VOSviewer were used. For each publication, the normalized citation score was calculated as the number of citations received by the publication divided by the average number of citations received by all publications from the same publication year within the analyzed dataset. The average normalized citation score was subsequently calculated for each country and author. A value of 1 indicates citation impact equal to the average of publications from the same year, whereas values greater than or less than 1 indicate citation impact above or below the corresponding annual average, respectively. Unless otherwise specified, the default settings of VOSviewer and CiteSpace were retained.

## 3 Results

### 3.1 Global research output: annual trends

A total of 1126 documents, consisting of 933 articles and 193 reviews, were retrieved from the WoSCC and Scopus for the period of January 1, 2006, to March 31, 2026. The annual publication volume on PFAS and cancer has increased substantially over this period (**Figure 2**). Following a period of steady growth from 2006 to 2021, research output accelerated sharply from 2022 onward, with a notable peak of 200 publications in 2025. This sharp growth indicates that the potential relationship between PFAS exposure and cancer has attracted increasing scholarly attention.

**Figure 2:**
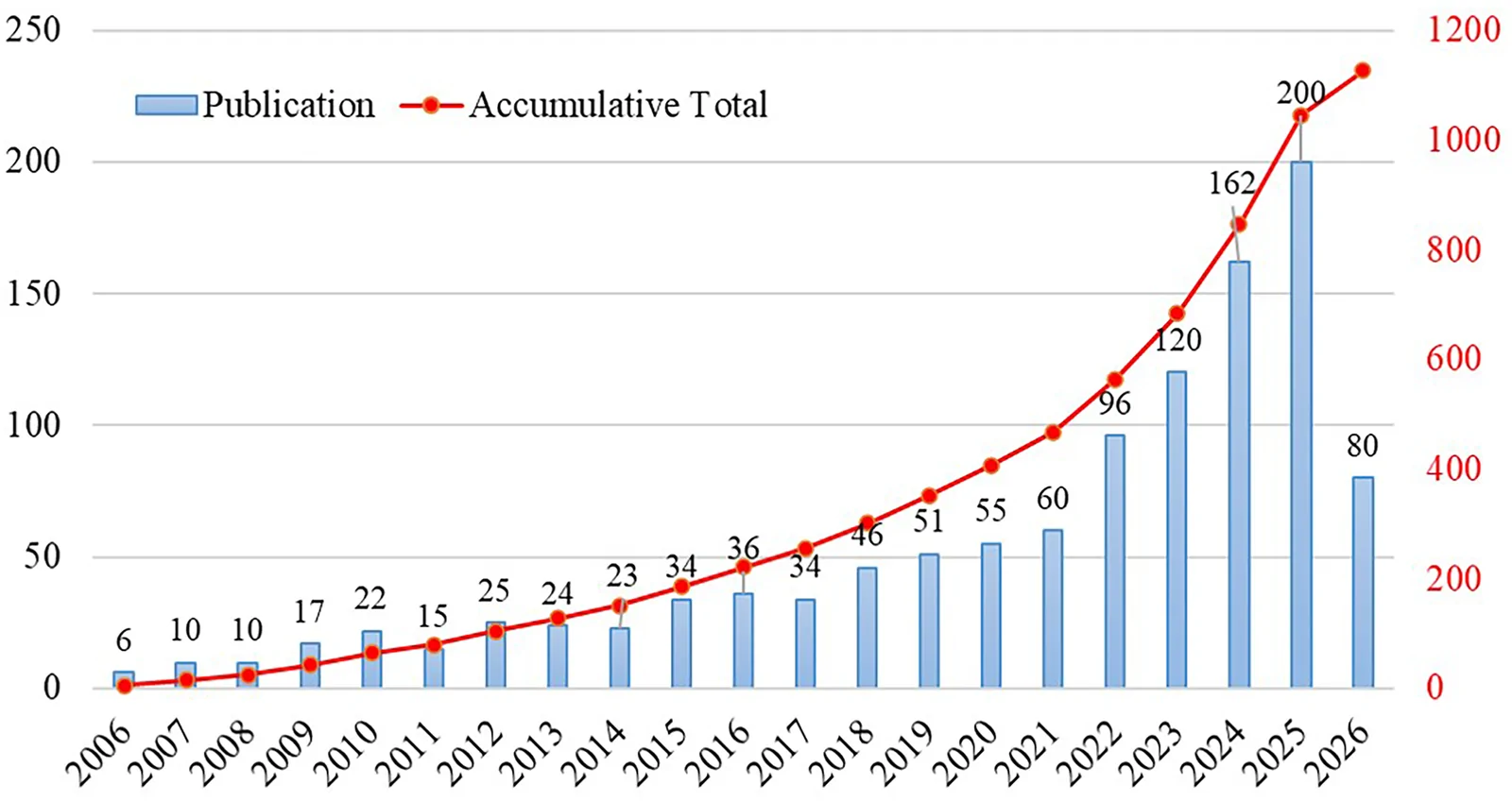
Annual and cumulative publication trends in global PFAS–cancer research.

### 3.2 National and institutional publication landscape

A total of 77 countries/regions contributed to research on PFAS and cancer. As shown in **Table 1**, the United States was the most productive country, with 451 publications, followed by China with 356 publications. All other countries contributed fewer than 100 publications, indicating that the United States and China accounted for a substantial proportion of the research output in this field. The United States also had the highest total link strength (205), followed by China (112) and England (73), demonstrating their relatively strong connectivity within the international collaboration network.

**Table 1:** Top 10 Countries for publications.

Rank	Country	Publications	Citations	Total link strength	Average citations per publication	Average normalized citations
1	USA	451	24911	205	55	1.1870
2	China	356	8831	112	25	0.8533
3	Italy	56	2082	42	37	0.9533
4	England	41	5524	73	135	1.9844
5	Sweden	39	2565	46	66	1.3494
6	Germany	38	2743	49	72	1.3168
7	Japan	36	1933	35	54	1.1439
8	Canada	35	1337	41	38	0.7194
9	Denmark	35	2251	48	64	1.0976
10	India	34	2098	39	62	1.3533

Citation performance was further evaluated using the average normalized citation score to reduce the influence of publication age. Among the 10 most productive countries, England had the highest average normalized citation score (1.9844), followed by India (1.3533), Sweden (1.3494), and Germany (1.3168). The United States also had an above-average normalized citation score (1.1870), whereas China had a score below the overall annual average (0.8533). These findings indicate that England demonstrated the highest year-normalized citation performance among the 10 most productive countries, despite its comparatively smaller publication output.

At the institutional level, 430 organizations contributed to PFAS–cancer research (**Figure 3**). The institutional collaboration network comprised 430 nodes and 1,135 links, with a network density of 0.0123. The largest connected component contained 281 institutions, accounting for 65% of all nodes. Thus, nearly two-thirds of the institutions were connected within the principal collaboration network. However, the relatively low network density indicates that interinstitutional collaboration remained generally sparse, suggesting considerable potential for strengthening collaboration among currently weakly connected or isolated institutions.

**Figure 3:**
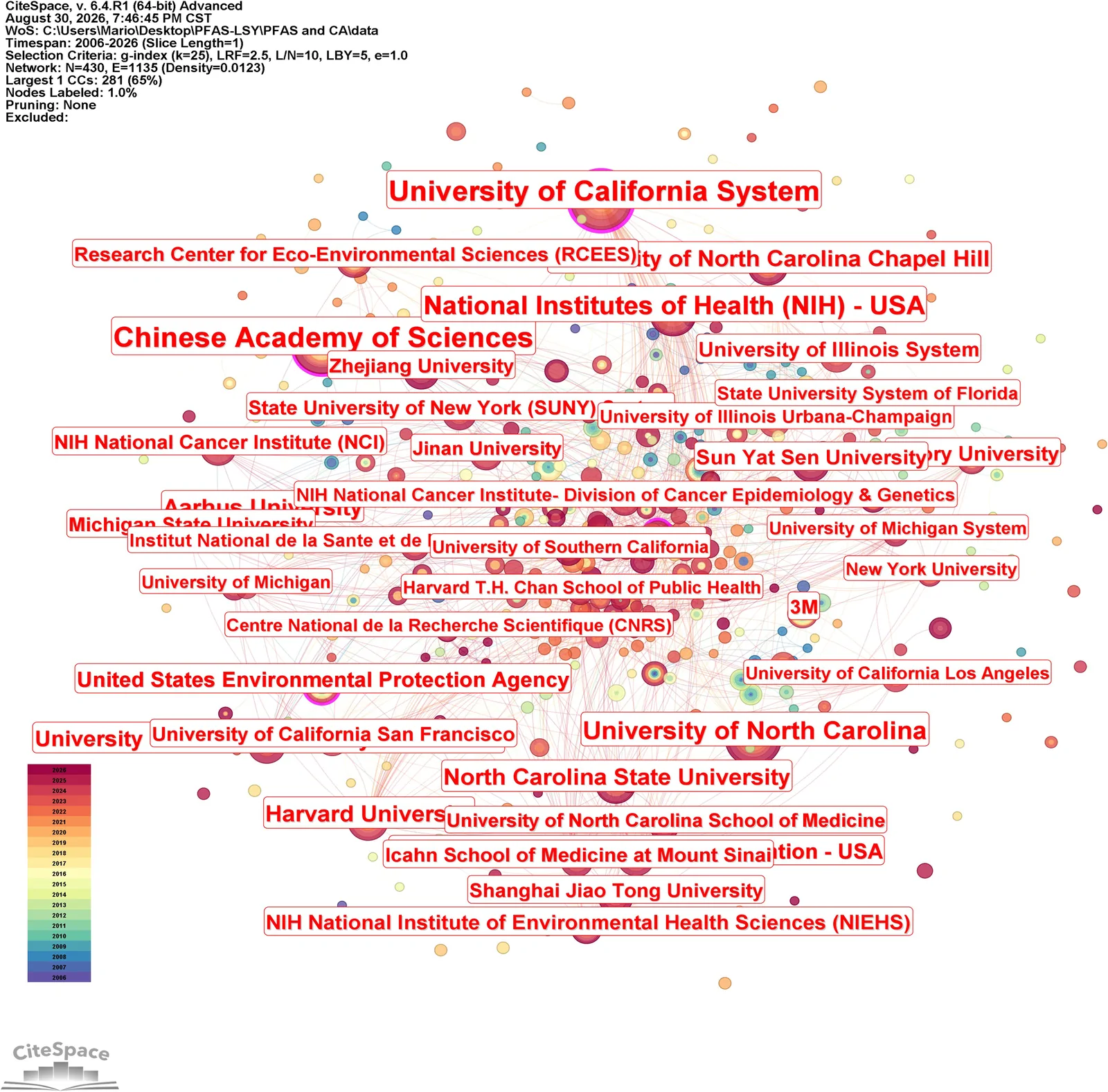
Analysis of institutional publications. Node size represents institutional publication output, links indicate collaborative relationships, and node colors correspond to publication years.

As shown in **Table 2**, the Chinese Academy of Sciences was the most productive institution, with 58 publications, followed by the University of California System (n = 55), the National Institutes of Health in the United States (n = 40), and the University of North Carolina (n = 35). The remaining institutions in the top 10 were Aarhus University (n = 25), Harvard University (n = 24), North Carolina State University (n = 23), the University of North Carolina at Chapel Hill (n = 23), the University of Chinese Academy of Sciences (n = 21), and the Centers for Disease Control and Prevention in the United States (n = 19). Of the 10 most productive institutions, seven were located in the United States, two in China, and one in Denmark. These findings indicate that institutions from the United States accounted for the largest proportion of the most productive organizations, while the Chinese Academy of Sciences had the highest individual institutional output.

**Table 2:** Top 10 institutions for publications.

Rank	Institution	Country	Documents
1	Chinese Academy of Sciences	China	58
2	University of California System	USA	55
3	National Institutes of Health (NIH)-USA	USA	40
4	University of North Carolina	USA	35
5	Aarhus University	Denmark	25
6	Harvard University	USA	24
7	North Carolina State University	USA	23
8	University of North Carolina Chapel Hill	USA	23
9	University of Chinese Academy of Sciences	China	21
10	Centers for Disease Control & Prevention - USA	USA	19

### 3.3 Analysis of journals

The 1126 publications were distributed among 373 distinct academic journals (**Figure 4**). *Environment International* (50 publications), *Environmental Research* (47 publications), *Journal of Hazardous Materials* (35 publications) and *Science of the Total Environment* (35 publications) were the most prolific (**Table 3**). Notably, the most productive journals are predominantly in the fields of environmental science and toxicology. All of the top ten journals are classified in the JCR Q1 quadrant, indicating that research on this topic is primarily published in high-impact journals within these disciplines.

**Figure 4:**
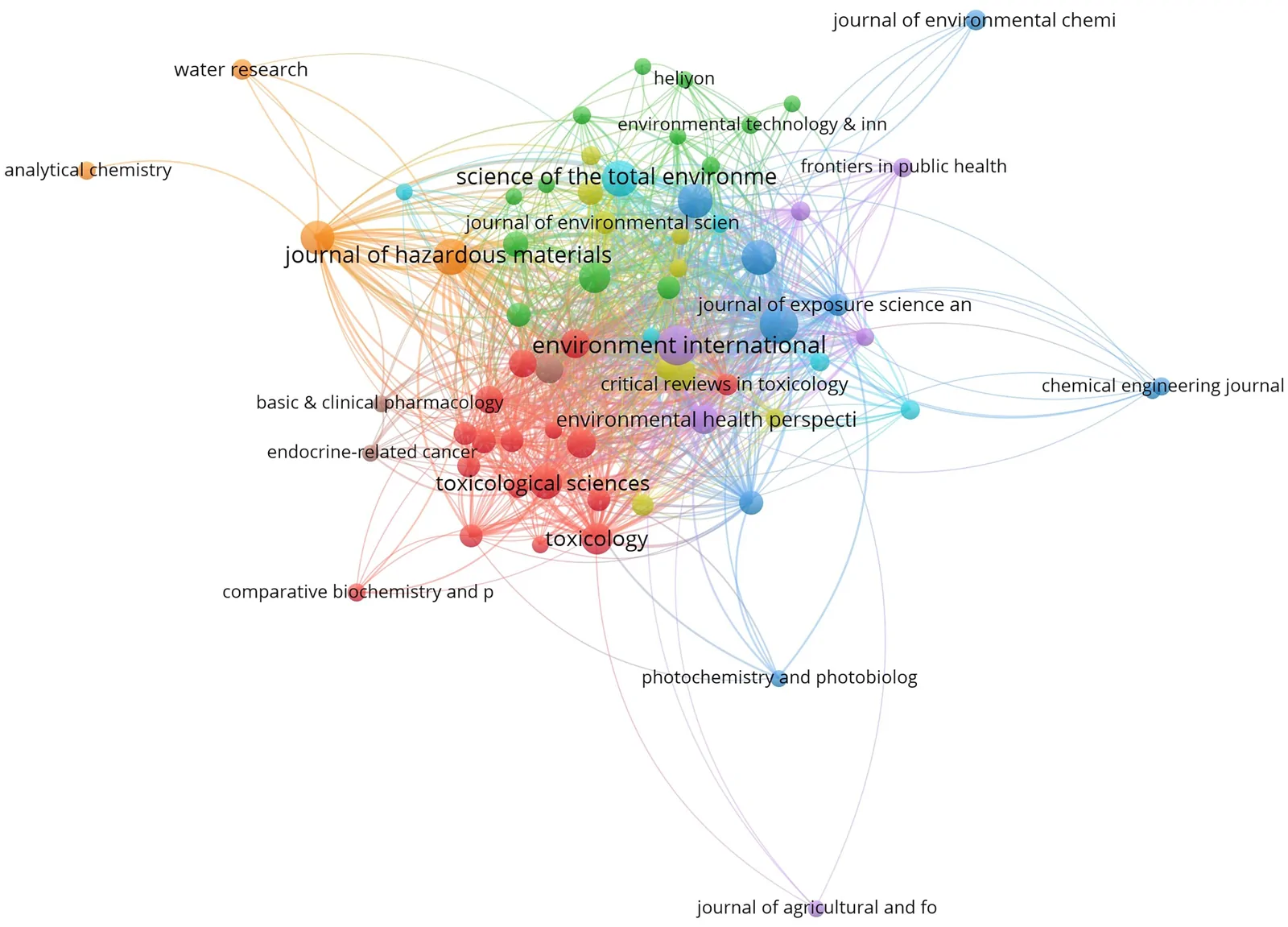
Analysis of journal publications. Node size represents journal publication output, links indicate coauthorship relationships, and node colors correspond to publication years.

**Table 3:** Top 10 journals for publications.

Rank	Journal	Documents	Total link strength	JCR
1	*Environment International*	50	469	Q1
2	*Environmental Research*	47	558	Q1
3	*Journal of Hazardous Materials*	35	221	Q1
4	*Science of the Total Environment*	35	202	Q1
5	*Ecotoxicology and Environmental Safety*	33	196	Q1
6	*Environmental Pollution*	32	225	Q1
7	*Environmental Science & Technology*	29	180	Q1
8	*Toxicological Sciences*	26	219	Q1
9	*Toxics*	26	188	Q1
10	*Chemosphere*	24	185	Q1

### 3.4 Author impact analysis

As detailed in **Table 4**, eight authors published at least 10 articles in the field of PFAS and cancer. Suzanne E. Fenton was the most productive author, with 18 publications, followed by Jiayin Dai (n = 12), Eva C. Bonefeld-Jørgensen (n = 11), Manhai Long (n = 11), and Geary W. Olsen (n = 11). Antonia M. Calafat, Lianghong Guo, and Kyle Steenland each published 10 articles. To reduce the influence of publication age on citation-based comparisons, author citation performance was evaluated using the average normalized citation score. Joseph G. Allen had the highest average normalized citation score (8.1395), followed by Jamie C. DeWitt (6.7978), Elsie M. Sunderland (6.6037), David V. Conti (5.2991), Alan Ducatman (4.6401), Christopher S. Lau (4.3809), Max Aung (4.3298), and Rob McConnell (4.2240). These authors demonstrated relatively high year-normalized citation performance within the analyzed dataset, although they were not necessarily the most prolific authors. As presented in **Table 5**, the geographic distribution of the top eight most productive authors is dominated by researchers from the United States, followed by a notable contingent from China, and also includes authors from Denmark. Specifically, the two Danish authors (Eva C Bonefeld-Jørgensen and Manhai Long) are both affiliated with Aarhus University.

**Table 4:** Most productive authors and authors with the highest average normalized citation scores.

Rank	Author	Documents	Rank	Author	Average normalized citations
1	Suzanne E Fenton	18	1	Joseph G Allen	8.1395
2	Jiayin Dai	12	2	Jamie C DeWitt	6.7978
3	Eva C Bonefeld-Jørgensen	11	3	Elsie M Sunderland	6.6037
4	Manhai Long	11	4	David V Conti	5.2991
5	Geary W Olsen	11	5	Alan Ducatman	4.6401
6	Antonia M Calafat	10	6	Christopher S Lau	4.3809
7	Lianghong Guo	10	7	Max Aung	4.3298
8	Kyle Steenland	10	8	Rob McConnell	4.224

**Table 5:** The geographic distribution of the most productive authors.

Rank	Author	Affiliation	Country
1	Suzanne E Fenton	National Institute of Environmental Health Sciences	USA
2	Jiayin Dai	Chinese Academy of Sciences	China
3	Eva C Bonefeld-Jørgensen	Aarhus University	Denmark
4	Manhai Long	Aarhus University	Denmark
5	Geary W Olsen	3M Company	USA
6	Antonia M Calafat	Centers for Disease Control and Prevention	USA
7	Lianghong Guo	University of the Chinese Academy of Sciences	China
8	Steenland, Kyle	Emory University	USA

### 3.5 Analysis of keywords

Based on the 1,126 included publications, 490 keywords were identified and included in the keyword co-occurrence analysis (**Figure 5**). The network comprised 490 nodes and 803 links, with a density of 0.0067. The largest connected component contained 292 nodes, accounting for 59% of all keywords. The low network density indicates that, although a principal group of interrelated research topics was present, the overall keyword network remained relatively dispersed.

**Figure 5:**
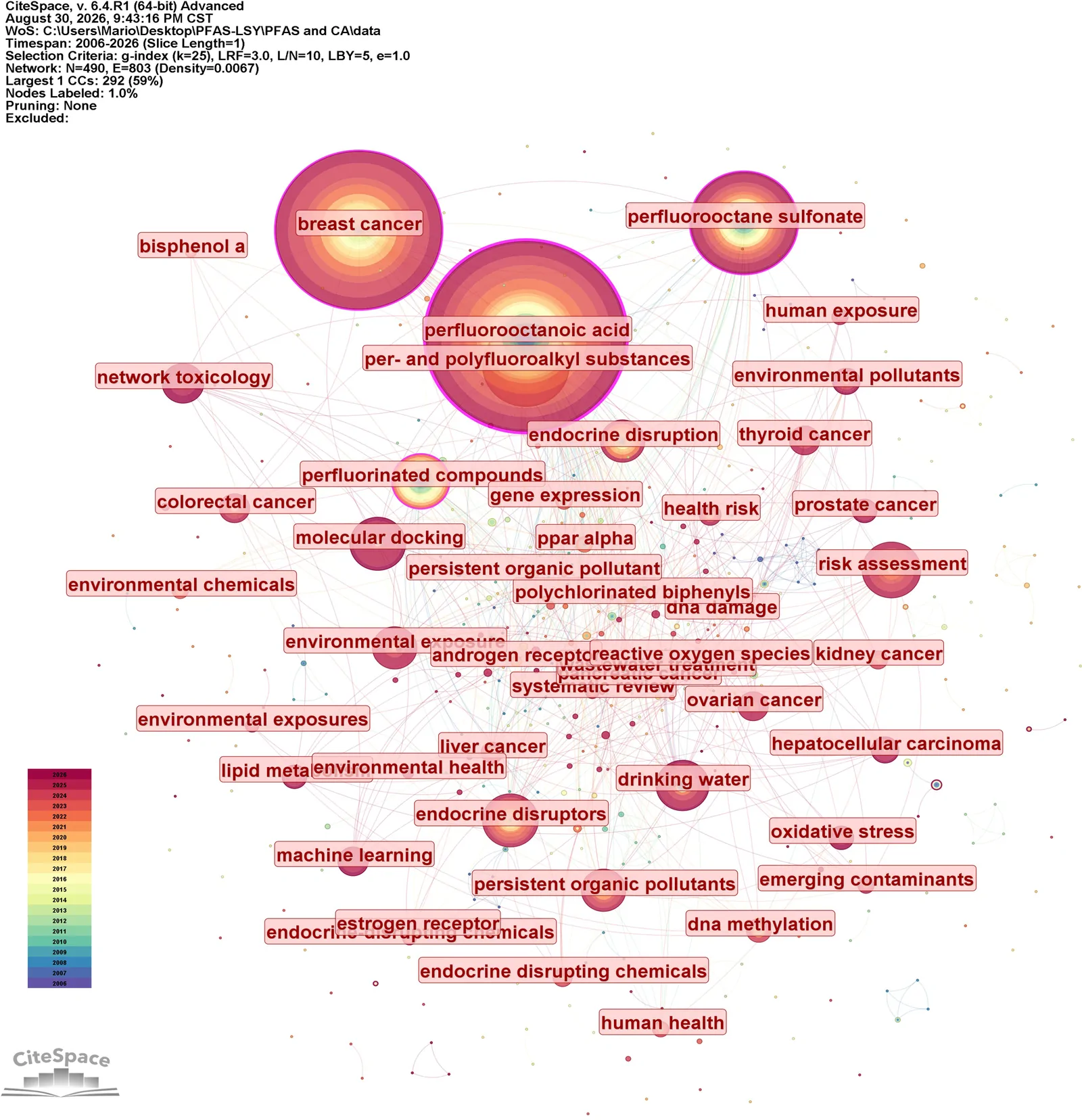
Keyword co-occurrence network in PFAS–cancer research. Links indicate co-occurrence relationships, and node colors correspond to publication years.

As shown in **Table 6**, 20 keywords occurred at least 10 times. The most frequent keyword was “per- and polyfluoroalkyl substances” (n = 116), followed by “perfluorooctanoic acid” (n = 90), “breast cancer” (n = 59), “perfluorooctane sulfonate” (n = 51), and “perfluorinated compounds” (n = 23). Other frequently occurring keywords included “risk assessment” (n = 21), “molecular docking” (n = 20), “endocrine disruptors” (n = 20), “drinking water” (n = 19), “endocrine disruption” (n = 16), “environmental exposure” (n = 16), “persistent organic pollutants” (n = 16), “perfluoroalkyl acids” (n = 16), and “network toxicology” (n = 15). Among the cancer-specific terms, “breast cancer” occurred most frequently, followed by “colorectal cancer”, “thyroid cancer”, and “ovarian cancer” (n = 11 each), and “hepatocellular carcinoma” (n = 10). The coexistence of exposure-related, cancer-related, and methodological keywords indicates that the literature encompasses exposure assessment, specific cancer sites, toxicological processes, and computational approaches.

**Table 6:** Keywords occurring at least 10 times in PFAS–cancer research.

Rank	Keyword	Frequency
1	per- and polyfluoroalkyl substances	116
2	perfluorooctanoic acid	90
3	breast cancer	59
4	perfluorooctane sulfonate	51
5	perfluorinated compounds	23
6	risk assessment	21
7	molecular docking	20
8	endocrine disruptors	20
9	drinking water	19
10	endocrine disruption	16
11	environmental exposure	16
12	persistent organic pollutants	16
13	perfluoroalkyl acids	16
14	network toxicology	15
15	machine learning	11
16	colorectal cancer	11
17	thyroid cancer	11
18	ovarian cancer	11
19	environmental pollutants	10
20	hepatocellular carcinoma	10

Keyword cluster analysis was subsequently conducted to characterize the thematic structure of the literature (**Figure 6**). The clustering network contained 361 nodes and 694 links, with a density of 0.0107. Ten keyword clusters were identified. The modularity Q value was 0.6245, indicating a clearly differentiated cluster structure, while the weighted mean silhouette score was 0.8733, suggesting high internal consistency and satisfactory reliability of the clustering solution. The harmonic mean of modularity and silhouette was 0.7282. The principal clusters included “breast cancer”, “polyfluoroalkyl substances”, “perfluorooctanoic acid”, “perfluorooctane sulfonate”, “endocrine-disrupting chemicals”, “emerging contaminants”, “network toxicology”, and “thyroid cancer”. Collectively, these clusters show that the knowledge structure of PFAS–cancer research was organized around specific PFAS compounds, cancer types, endocrine-related effects, emerging contaminants, and computational toxicology.

**Figure 6:**
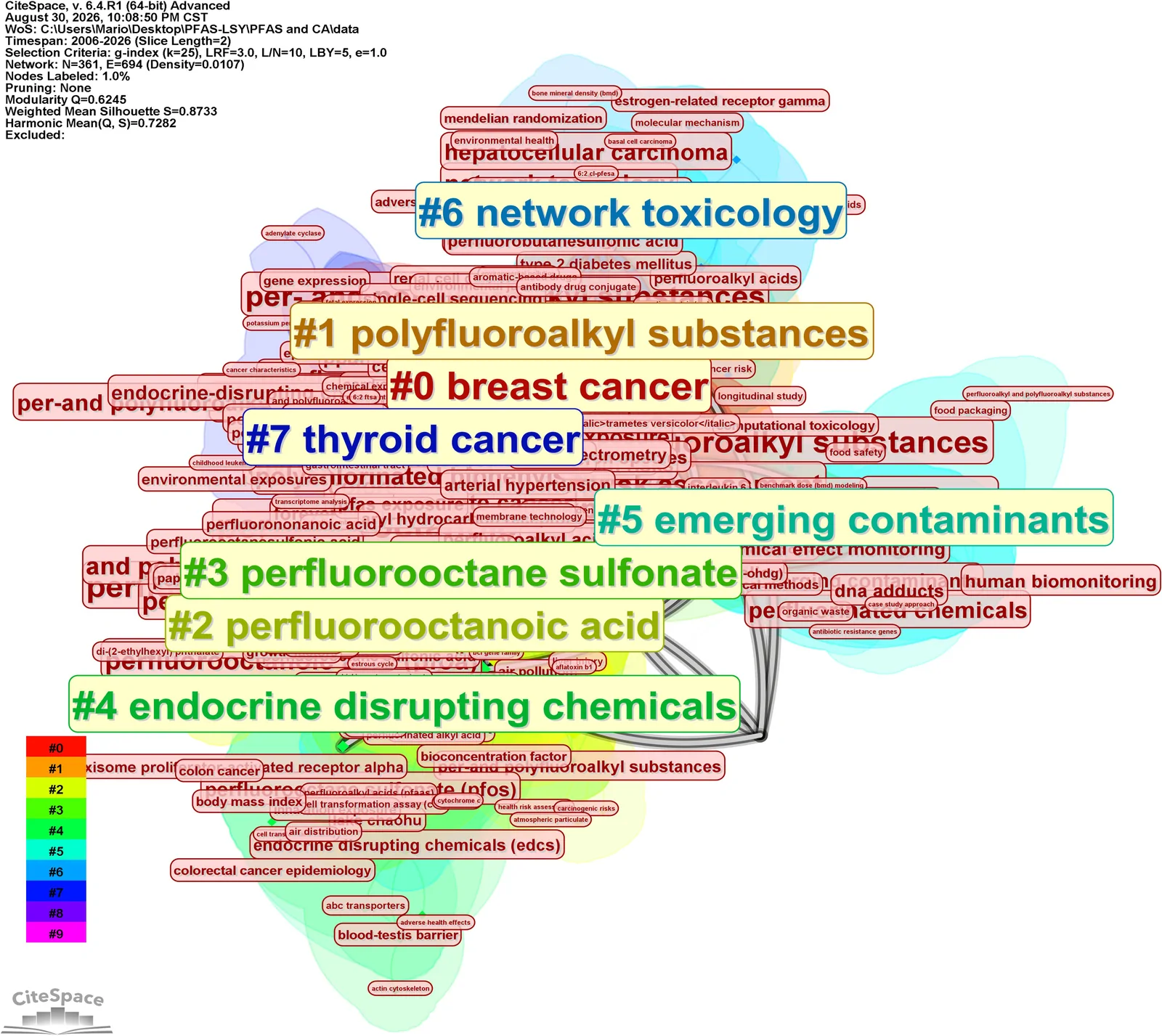
Keyword clustering network in PFAS–cancer research. Colors represent different thematic clusters labeled using the log-likelihood ratio method, and cluster numbers are assigned in descending order of cluster size.

Citation-burst analysis identified the 10 keywords with the strongest bursts during 2006–2026 (**Figure 7**). “Network toxicology” exhibited the highest burst strength (6.74), followed by “molecular docking” (6.20) and “machine learning” (4.93); all three bursts began in 2025 and continued through 2026. “Per- and polyfluoroalkyl substances” showed the longest ongoing burst, beginning in 2021 and continuing through 2026, with a burst strength of 3.71. “Thyroid cancer” showed a sustained burst from 2023 to 2026 (strength = 3.06), while “environmental exposure” (strength = 3.17), “lipid metabolism” (strength = 2.89), and “ovarian cancer” (strength = 2.69) showed bursts from 2024 to 2026. Earlier bursts included “perfluoroalkyl acids” from 2021 to 2022 (strength = 3.38) and “risk assessment” from 2022 to 2024 (strength = 2.82). These findings indicate that computational toxicology, machine learning, exposure assessment, metabolic processes, and selected cancer types have received rapidly increasing research attention in recent years.

**Figure 7:**
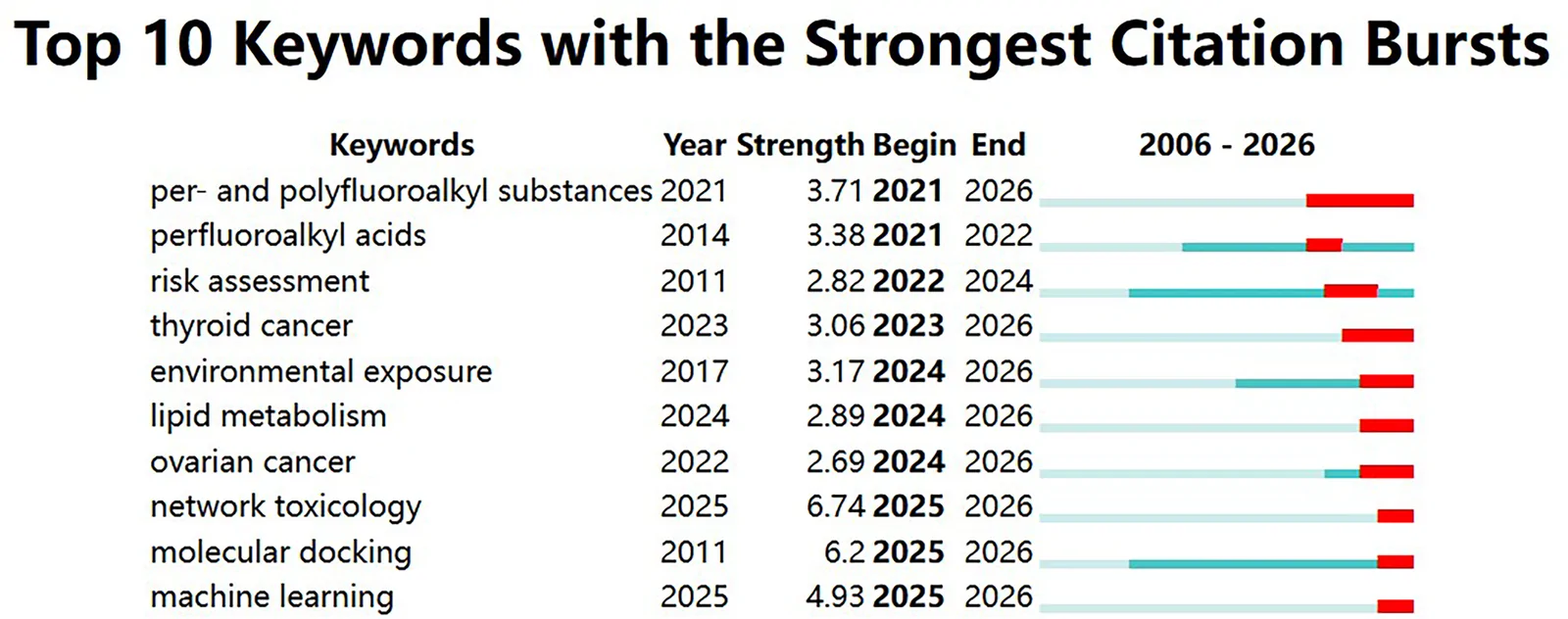
Top 10 keywords with the strongest citation bursts. Red segments indicate the duration of citation bursts, while burst strength represents the intensity of the increase in scholarly attention.

The keyword timeline visualization further illustrated the temporal evolution of research topics (**Figure 8**). Earlier studies were primarily centered on legacy PFAS compounds, particularly perfluorooctanoic acid and perfluorooctane sulfonate, together with breast cancer, developmental toxicity, drinking water, gene expression, and PPARα-related research. Subsequent studies expanded toward risk assessment, endocrine disruption, environmental exposure, colorectal cancer, hepatocellular carcinoma, DNA damage, estrogen- and androgen-receptor pathways, and persistent organic pollutants. In the most recent period, the literature increasingly included a broader range of cancer types, such as thyroid, ovarian, prostate, pancreatic, testicular, and renal cell cancers, as well as topics including lipid metabolism, PI3K/Akt/mTOR signaling, single-cell sequencing, network toxicology, molecular docking, and machine learning. The convergence of the timeline and citation-burst results suggests that computational approaches, emerging analytical methods, metabolic pathways, and broader cancer-site assessment represent areas of increasing research activity.

**Figure 8:**
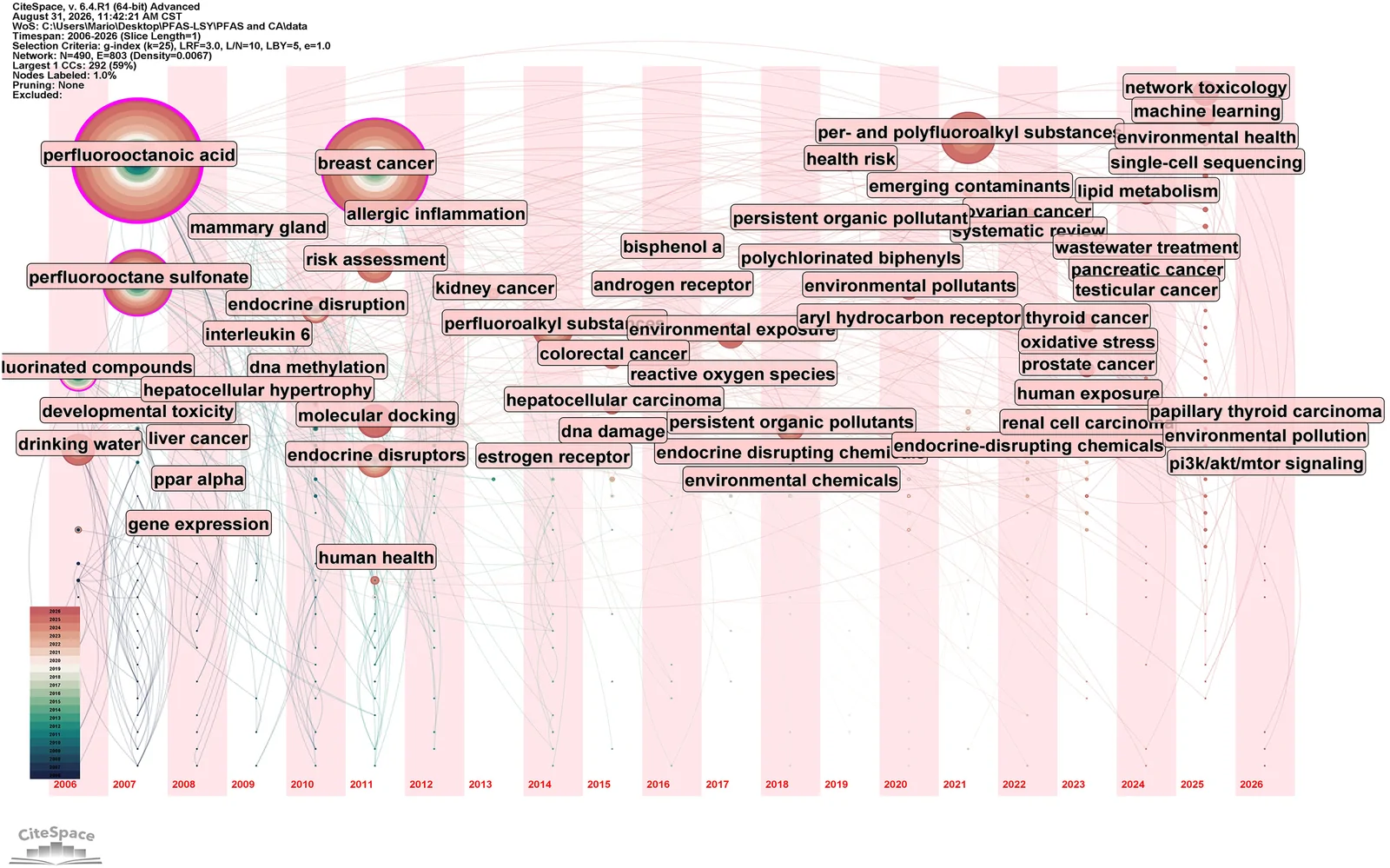
Timeline visualization of keyword co-occurrence in PFAS–cancer research. Keywords are arranged chronologically within their respective clusters; links indicate co-occurrence relationships.

## 4 Discussion

PFAS have emerged as global environmental contaminants of great concern, primarily due to their extreme persistence, bioaccumulation potential, and multi-organ toxicity. Biomonitoring data have consistently shown that human exposure is widespread; for example, a U.S. National Health and Nutrition Examination Survey (NHANES) study found detectable serum levels of PFAS in over 98% of the U.S. population (16). Regulatory and cancer-hazard evaluations have also evolved. In 2019, PFOA, its salts, and PFOA-related compounds were listed in Annex A of the Stockholm Convention, subject to specified exemptions. More recently, the International Agency for Research on Cancer classified PFOA as carcinogenic to humans (Group 1) and PFOS as possibly carcinogenic to humans (Group 2B). These developments have coincided with increasing research attention to the potential relationship between PFAS exposure and cancer.

Consistent with this growing attention, our bibliometric analysis showed a marked increase in publications after 2022. Although bibliometric data cannot establish the causes of this growth, several scientific and regulatory developments may have contributed to the observed increase. First, the expanding body of epidemiological evidence from large-scale population studies has demonstrated associations between PFAS and various cancer types (17–19). The IARC’s recent reclassification of PFOA and PFOS was particularly influential in raising both scientific and public alarm. Second, due to their high stability and resistance to degradation, PFAS have achieved a ubiquitous presence globally, with detection even in remote areas and in the human blood of indigenous populations (20, 21). This widespread exposure creates an urgent need for researchers to understand their potential impact on global public health. Third, the bioaccumulative nature of PFAS causes them to build up in human tissues over time, raising the risk of adverse health outcomes (22). This cumulative toxic burden has spurred researchers to investigate the long-term consequences of this accumulation. Finally, advancements in analytical chemistry, such as high-sensitivity mass spectrometry (23), have enabled the precise quantification of low-dose, chronic exposure, which has been crucial for a more nuanced understanding of the dose-response relationship in humans.

The geographic analysis showed a marked concentration of publication output in the United States and China. The United States produced the largest number of publications and had the highest total link strength, followed by China, indicating that these two countries combined high research output with relatively extensive international collaboration. The United States boasts a large number of leading research institutions in this area. This may be closely linked to its long history of research on PFAS and the substantial data resources accumulated from major contamination events, such as The C8 Health Project (24). However, publication volume and network connectivity represent different dimensions of research activity and should not be interpreted as direct measures of study quality. After adjustment for publication year, England had the highest average normalized citation score among the 10 most productive countries, followed by India, Sweden, and Germany. This finding differs from the ranking based on absolute publication output and illustrates the importance of accounting for citation-accumulation time. Nevertheless, normalized citation scores remain measures of bibliometric visibility within the analyzed dataset and are influenced by database coverage, collaboration patterns, journal visibility, and citation practices; they should therefore not be equated with scientific quality or innovation.

Author-level analyses showed that research productivity and year-normalized citation performance represented distinct dimensions of scholarly activity. Suzanne E. Fenton was the most productive author, followed by Jiayin Dai, Eva C. Bonefeld-Jørgensen, Manhai Long, Geary W. Olsen, Antonia M. Calafat, Lianghong Guo, and Kyle Steenland. In contrast, Joseph G. Allen had the highest average normalized citation score (8.1395), followed by Jamie C. DeWitt (6.7978), Elsie M. Sunderland (6.6037), David V. Conti (5.2991), Alan Ducatman (4.6401), Christopher S. Lau (4.3809), Max Aung (4.3298), and Rob McConnell (4.2240). The limited overlap between the most productive and most highly normalized-cited authors indicates that a larger publication output did not necessarily correspond to greater citation visibility after adjustment for publication year. Among the eight most productive authors, four were affiliated with institutions in the United States, two in China, and two in Denmark. Notably, Eva C. Bonefeld-Jørgensen and Manhai Long were both affiliated with Aarhus University and coauthored several studies in this field (25–27), reflecting sustained institutional collaboration.

Analyzing where PFAS-cancer research is published is key, as researchers tend to submit to journals that are a strong fit. Our analysis found that most papers are concentrated in environmental science and toxicology journals. Importantly, All 10 of the most productive journals were classified in the JCR Q1 quartile, indicating that PFAS–cancer research was frequently published in highly ranked journals within environmental science and toxicology. However, journal-level rankings do not directly indicate the methodological quality or evidential strength of individual studies. Furthermore, we observed that an increasing number of related studies have also begun to appear in journals from other disciplines, such as endocrinology and chemistry. This reveals that the field has formed a multidisciplinary journal cluster, with environmental science and toxicology at its core. These publication patterns indicate that environmental science and toxicology constitute the principal disciplinary outlets for this research. Our findings can help guide other scholars on where to submit their work.

With over 14,000 different PFAS compounds identified, the research focus has evolved over time (28). In the early stages, “perfluorooctanoic acid” and “perfluorooctane sulfonate” were the most frequently appearing keywords, reflecting the initial emphasis on these two ubiquitous compounds. However, as these traditional PFAS are phased out and new alternatives emerge, the research focus has shifted. Researchers are now investigating the health effects of these newer PFAS replacement chemicals, rather than just PFOA and PFOS (29, 30). This pattern reflects increasing research attention to the potential health effects of emerging and replacement PFAS.

Similarly, the investigation into cancer types has also expanded. While “breast cancer” has the most research articles, likely due to its high incidence and the role PFAS can play as endocrine disruptors (31), the field is broadening its scope. Research is moving from its early, concentrated focus on breast cancer to a wider exploration of multiple cancer types. This expansion reflects a diversification of research attention across cancer types rather than confirmation of associations between PFAS exposure and these malignancies. Future studies should evaluate these potential associations and investigate their biological plausibility using rigorous epidemiological and experimental designs.

The keyword co-occurrence and clustering analyses revealed that PFAS–cancer research encompasses several interconnected themes, including specific PFAS compounds, cancer types, endocrine-disrupting effects, exposure assessment, emerging contaminants, and computational toxicology. The clustering solution showed satisfactory structural quality, with a modularity Q value of 0.6245 and a weighted mean silhouette score of 0.8733, indicating clear separation and high internal consistency among the identified thematic clusters. The high frequencies of “per- and polyfluoroalkyl substances”, “perfluorooctanoic acid”, “perfluorooctane sulfonate”, and “breast cancer” reflect the sustained attention given to legacy PFAS and selected cancer sites. Meanwhile, clusters related to emerging contaminants and network toxicology suggest that the field has expanded beyond individual legacy compounds toward broader chemical classes, alternative compounds, and computational approaches.

“Environmental exposure” exhibited a recent citation burst with a strength of 3.17 from 2024 to 2026, indicating increasing attention to PFAS exposure pathways, exposure levels, and the health implications of occupational and chronic low-dose exposure (28, 32). Accurately measuring exposure is a top priority and a major challenge for risk assessment. For instance, PFOA has a long biological half-life of 1.48 to 5.1 years (33). Due to its bioaccumulation potential, serum PFOA levels are considered a reliable biomarker for assessing long-term exposure and the total body burden, which is crucial for evaluating potential health risks (11, 34). Serum PFAS levels are considered a dependable measure of cumulative exposure from all sources. Unlike environmental monitoring, which only estimates external exposure (e.g., via air, water, or soil), biomonitoring provides a comprehensive assessment of actual internal exposure from all sources, including food, water, air, and consumer products (34). Therefore, it is a fundamental tool for evaluating the relationship between PFAS body burden and adverse health outcomes like cancer. Currently, mass spectrometry is currently the most reliable and commonly used method for assessing human serum PFAS levels (35, 36). This technology provides solid data for studies and helps us understand the toxicology and public health risks of PFAS, despite a few remaining challenges. Its progress has been vital for advancing research in this area.

The combined citation-burst and timeline analyses showed increasing research attention to computational methods and selected molecular processes. “Network toxicology” displayed the strongest citation burst (strength = 6.74), followed by “molecular docking” (6.20) and “machine learning” (4.93), with all three bursts beginning in 2025 and continuing through 2026. Their simultaneous appearance in the recent portion of the keyword timeline suggests a growing use of computational approaches to prioritize PFAS compounds, predict molecular targets, and generate hypotheses regarding potential toxicity pathways (37). “Lipid metabolism” also showed a recent citation burst from 2024 to 2026 (strength = 2.89) and appeared in the latest portion of the timeline, providing convergent evidence that metabolic perturbation is an emerging area of research activity. Recent multi-omics and computational studies have further explored potential associations between PFAS exposure, lipid and glucose metabolic signaling, and tumor-related processes (38). By contrast, “oxidative stress” appeared as a continuing mechanistic theme in the co-occurrence and timeline analyses but did not exhibit a recent citation burst. Oxidative stress has long been investigated as a potential component of PFAS toxicity because excessive reactive oxygen species may disrupt cellular redox homeostasis and contribute to oxidative damage to lipids, proteins, and DNA (39). Nevertheless, its bibliometric prominence does not establish a causal role in PFAS-related carcinogenesis. Single-cell sequencing and PI3K/Akt/mTOR signaling appeared in the latest period of the timeline but were not among the strongest burst terms or independent major clusters; they should therefore be regarded as newly appearing or exploratory topics rather than established research hotspots. Overall, these bibliometric patterns reflect changes in scholarly attention and provide directions for hypothesis generation, but they do not establish causal mechanisms or confirm that the identified pathways represent therapeutic targets.

## 5 Limitations

The present study is subject to the following limitations. First, the dataset was derived exclusively from WoSCC and Scopus. Although these databases provide broad multidisciplinary coverage and relatively complete citation metadata, relevant publications indexed exclusively in PubMed/MEDLINE, Embase, regional databases, or non-English-language journals may have been omitted. Because WoSCC and Scopus favor internationally visible and English-language journals, the contributions of some countries, institutions, and regionally focused research communities may have been underestimated. Database-specific differences in journal coverage and citation indexing may also have influenced country rankings, citation indicators, and the apparent prominence of research hotspots. Therefore, these findings should be interpreted as patterns within the selected databases rather than as an exhaustive representation of global PFAS–cancer research. Future studies could incorporate additional databases and perform database-specific sensitivity analyses to assess the robustness of the results. Second, due to the inherent lag in citation accumulation, the citation visibility of recently published studies may not be fully reflected because of the time required for citations to accumulate. Future research is recommended to maintain ongoing attention to this issue and to update the dataset periodically to incorporate the latest scholarly findings.

## 6 Conclusions

This study provides a systematic bibliometric overview of the global research landscape on PFAS and cancer. A total of 1,126 publications were included, and research output increased markedly after 2022. The United States and China contributed the largest numbers of publications, with the United States showing the strongest international collaboration connectivity. At the institutional level, the Chinese Academy of Sciences had the highest publication output, while institutions from the United States accounted for seven of the 10 most productive organizations. Nevertheless, the low density of the institutional collaboration network indicates that interinstitutional cooperation remains relatively sparse. Publications were concentrated primarily in high-impact environmental science and toxicology journals. Keyword co-occurrence and clustering analyses showed that the field was organized around specific PFAS compounds, cancer types, endocrine-disrupting effects, exposure assessment, emerging contaminants, and computational toxicology. The temporal analysis indicated a broadening of research attention from legacy compounds, particularly PFOA and PFOS, toward the wider PFAS class and a more diverse range of cancer sites. Citation-burst and timeline analyses identified network toxicology, molecular docking, machine learning, and lipid metabolism as areas of rapidly increasing recent research activity. In contrast, oxidative stress represented a continuing mechanistic theme, while single-cell sequencing and PI3K/Akt/mTOR signaling appeared as newly emerging but still exploratory topics. These bibliometric patterns reflect changes in scholarly attention rather than confirmation of causal mechanisms or evidence quality. Future research should strengthen international and interinstitutional collaboration and integrate rigorous exposure assessment, epidemiological evidence, experimental validation, and multi-omics approaches to clarify the potential role of PFAS in carcinogenesis.

## References

[1] Bray F Laversanne M Sung H Ferlay J Siegel RL Soerjomataram I . Global cancer statistics 2022: GLOBOCAN estimates of incidence and mortality worldwide for 36 cancers in 185 countries. CA Cancer J Clin. (2024) 74:229–63. doi: 10.3322/caac.21834 38572751

[2] Sharma N Kumar V S V Umesh M Sharma P Thazeem B . Hazard identification of endocrine-disrupting carcinogens (EDCs) in relation to cancers in humans. Environ Toxicol Pharmacol. (2024) 109:104480. doi: 10.1016/j.etap.2024.104480 38825092

[3] van Gerwen M Colicino E Guan H Dolios G Nadkarni GN Vermeulen RCH . Per- and polyfluoroalkyl substances (PFAS) exposure and thyroid cancer risk. eBioMedicine. (2023) 97:104831. doi: 10.1016/j.ebiom.2023.104831 37884429 PMC10667111

[4] Goswami L Dikshit PK Prakash A Vidanage PW Verma JP Oh S . A critical review on occurrence, speciation, mobilization, and toxicity of per- and polyfluoroalkyl substances in the soil-microbe-plant system and bioremediation strategies. J Hazard Mater. (2025) 494:138743. doi: 10.1016/j.jhazmat.2025.138743 40450997

[5] Cousins IT Johansson JH Salter ME Sha B Scheringer M . Outside the safe operating space of a new planetary boundary for per- and polyfluoroalkyl substances (PFAS). Environ Sci Technol. (2022) 56:11172–9. doi: 10.1021/acs.est.2c02765 35916421 PMC9387091

[6] Crone BC Speth TF Wahman DG Smith SJ Abulikemu G Kleiner EJ . Occurrence of per- and polyfluoroalkyl substances (PFAS) in source water and their treatment in drinking water. Crit Rev Environ Sci Technol. (2019) 49:2359–96. doi: 10.1080/10643389.2019.1614848 32831535 PMC7433796

[7] Jusko TA Oktapodas M Murinová LP Babinská K Babjaková J Verner MA . Demographic, reproductive, and dietary determinants of perfluorooctane sulfonic (PFOS) and perfluorooctanoic acid (PFOA) concentrations in human colostrum. Environ Sci Technol. (2016) 50:7152–62. doi: 10.1021/acs.est.6b00195 27244128 PMC5256678

[8] Rhee J Chang VC Cheng IA Calafat AM Botelho JC Shearer JJ . Serum concentrations of per- and polyfluoroalkyl substances and risk of renal cell carcinoma in the Multiethnic Cohort Study. Environ Int. (2023) 180:108197. doi: 10.1016/j.envint.2023.108197 37741007 PMC11093414

[9] Fenton SE Ducatman A Boobis A DeWitt JC Lau C Ng C . Per- and polyfluoroalkyl substance toxicity and human health review: Current state of knowledge and strategies for informing future research. Environ Toxicol Chem. (2021) 40:606–30. doi: 10.1002/etc.4890 33017053 PMC7906952

[10] Zheng J Liu S Yang JJ Zheng SJ Sun BS . - and polyfluoroalkyl substances (PFAS) and cancer: Detection methodologies, epidemiological insights, potential carcinogenic mechanisms, and future perspectives. Sci Total Environ. (2024) 953:176158. doi: 10.1016/j.scitotenv.2024.176158 39255941

[11] Spyrakis F Tiburtini GA Bruno S Dragani TA Colombo F . PFOA biomonitoring and kidney cancer risk: a meta-analysis of serum levels. Front Oncol. (2025) 15:1593300. doi: 10.3389/fonc.2025.1593300 40809024 PMC12344524

[12] Sassano M Seyyedsalehi MS Kappil EM Zhang SR Zheng TZ Boffetta P . Exposure to per- and poly-fluoroalkyl substances and lung, head and neck, and thyroid cancer: A systematic review and meta-analysis. Environ Res. (2025) 266:120606. doi: 10.1016/j.envres.2024.120606 39672496

[13] Drury NL Prueitt RL Beck BD . Commentary: Understanding IARC's PFOA and PFOS carcinogenicity assessments. Regul Toxicol Pharm. (2024) 154:105726. doi: 10.1016/j.yrtph.2024.105726 39433235

[14] Lin X Zhang GQ Cao SH Wang SL Wang F Xu M . A bibliometric analysis of wearable device research in sleep science: Trends and hotspots. Medicine. (2025) 104:e42853. doi: 10.1097/MD.0000000000042853 40527826 PMC12173289

[15] Montazeri A Mohammadi S Hesari PM Ghaemi M Riazi H Sheikhi-Mobarakeh Z . Preliminary guideline for reporting bibliometric reviews of the biomedical literature (BIBLIO): a minimum requirements. Syst Rev-London. (2023) 12:239. doi: 10.1186/s13643-023-02410-2 38102710 PMC10722750

[16] Calafat AM Kato K Hubbard K Jia T Botelho JC Wong LY . Legacy and alternative - and polyfluoroalkyl substances in the US general population: Paired serum-urine data from the 2013-2014 National Health and Nutrition Examination Survey. Environ Int. (2019) 131:105048. doi: 10.1016/j.envint.2019.105048 31376596 PMC7879379

[17] Shahi S Winquist A Troeschel AN Diver WR Hodge JM Deubler E . A case-cohort study of the association between per- and polyfluoroalkyl substances (PFAS) and breast cancer among participants in the American Cancer Society's Cancer Prevention Study-II. Environ Res. (2025) 285:122381. doi: 10.1016/j.envres.2025.122381 40692028

[18] Mancini FR Cano-Sancho G Gambaretti J Marchand P Boutron-Ruault MC Severi G . Perfluorinated alkylated substances serum concentration and breast cancer risk: Evidence from a nested case-control study in the French E3N cohort. Int J Cancer. (2020) 146:917–28. doi: 10.1002/ijc.32357 31008526

[19] Purdue MP Rhee J Denic-Roberts H McGlynn KA Byrne C Sampson J . A nested case-control study of serum per- and polyfluoroalkyl substances and testicular germ cell tumors among US Air Force servicemen. Environ Health Perspect. (2023) 131:077007. doi: 10.1289/Ehp12603 37458713 PMC10351502

[20] Sonne C Desforges JP Gustavson K Bossi R Bonefeld-Jorgensen EC Long MH . Assessment of exposure to perfluorinated industrial substances and risk of immune suppression in Greenland and its global context: a mixed-methods study. Lancet Planet Health. (2023) 7:E570–9. doi: 10.1016/S2542-5196(23)00106-7 37437998

[21] Hartz WF Björnsdotter MK Yeung LWY Humby JD Eckhardt S Evangeliou N . Sources and seasonal variations of per- and polyfluoroalkyl substances (PFAS) in surface snow in the Arctic. Environ Sci Technol. (2024) 58:21817–28. doi: 10.1021/acs.est.4c08854 39588978 PMC11636200

[22] Domingo JL . A review of the occurrence and distribution of per- and polyfluoroalkyl substances (PFAS) in human organs and fetal tissues. Environ Res. (2025) 272:121181. doi: 10.1016/j.envres.2025.121181 39978621

[23] Chang VC Rhee J Berndt SI Moore SC Freedman ND Jones RR . Serum perfluorooctane sulfonate and perfluorooctanoate and risk of postmenopausal breast cancer according to hormone receptor status: An analysis in the Prostate, Lung, Colorectal and Ovarian Cancer Screening Trial. Int J Cancer. (2023) 153:775–82. doi: 10.1002/ijc.34487 36843273 PMC10405832

[24] Frisbee SJ Brooks AP Maher A Flensborg P Arnold S Fletcher T . The C8 Health Project: Design, methods, and participants. Environ Health Perspect. (2009) 117:1873–82. doi: 10.1289/ehp.0800379 20049206 PMC2799461

[25] Ghisari M Eiberg H Long M Bonefeld-Jorgensen EC . Polymorphisms in Phase I and Phase II genes and breast cancer risk and relations to persistent organic pollutant exposure: a case-control study in Inuit women. Environ Health-Glob. (2014) 13:19. doi: 10.1186/1476-069x-13-19 24629213 PMC4234380

[26] Bonefeld-Jorgensen EC Long MH Fredslund SO Bossi R Olsen J . Breast cancer risk after exposure to perfluorinated compounds in Danish women: a case-control study nested in the Danish National Birth Cohort. Cancer Cause Control. (2014) 25:1439–48. doi: 10.1007/s10552-014-0446-7 25148915 PMC4215104

[27] Ghisari M Long M Roge DM Olsenb J Bonefeld-Jorgensen EC . Polymorphism in xenobiotic and estrogen metabolizing genes, exposure to perfluorinated compounds and subsequent breast cancer risk: A nested case-control study in the Danish National Birth Cohort. Environ Res. (2017) 154:325–33. doi: 10.1016/j.envres.2017.01.020 28157646

[28] Choi MA Rose S Langouet S . Per- and polyfluoroalkyl substances as potentiators of hepatotoxicity in an exposome framework: Current challenges of environmental toxicology. Toxicology. (2025) 515:154167. doi: 10.1016/j.tox.2025.154167 40300710

[29] Cao HM Zhou Z Wang L Liu GL Sun YZ Wang YW . Screening of potential PFOS alternatives to decrease liver bioaccumulation: Experimental and computational approaches. Environ Sci Technol. (2019) 53:2811–9. doi: 10.1021/acs.est.8b05564 30735364

[30] Chang CJ Young AS Keil A Mullins CE Liang DH Zhao SS . Novel and legacy per- and polyfluoroalkyl substances in humans: Long-term temporal variability and metabolic perturbations. Environ Int. (2025) 201:109590. doi: 10.1016/j.envint.2025.109590 40516284 PMC12219314

[31] Wan MLY Co VA El-Nezami H . Endocrine disrupting chemicals and breast cancer: a systematic review of epidemiological studies. Crit Rev Food Sci Nutr. (2022) 62:6549–76. doi: 10.1080/10408398.2021.1903382 33819127

[32] He AE Liang Y Li J Zhou Z Li FF Li Z . A critical review of populations with occupational exposure to per- and polyfluoroalkyl substances: External exposome, internal exposure levels, and health effects. Environ Sci Technol. (2025) 59:10715–33. doi: 10.1021/acs.est.4c14478 40442988

[33] Rosato I Bonato T Fletcher T Batzella E Canova C . Estimation of per- and polyfluoroalkyl substances (PFAS) half-lives in human studies: a systematic review and meta-analysis. Environ Res. (2024) 242:117743. doi: 10.1016/j.envres.2023.117743 38008199

[34] Gomis MI Vestergren R MacLeod M Mueller JF Cousins IT . Historical human exposure to perfluoroalkyl acids in the United States and Australia reconstructed from biomonitoring data using population-based pharmacokinetic modelling. Environ Int. (2017) 108:92–102. doi: 10.1016/j.envint.2017.08.002 28818713

[35] Belay MH Robotti E Ghignone A Fabbris A Brandi J Cecconi D . Sensitive and accurate determination of 32 PFAS in human serum using online SPE-UHPLC-HRMS. J Hazard Mater. (2025) 485:136780. doi: 10.1016/j.jhazmat.2024.136780 39667152

[36] Wang F Lin YX Qin L Zeng XT Jiang HC Liang YL . Serum metabolome associated with novel and legacy per- and polyfluoroalkyl substances exposure and thyroid cancer risk: A multi-module integrated analysis based on machine learning. Environ Int. (2025) 195:109203. doi: 10.1016/j.envint.2024.109203 39673872

[37] Hong Y Wang D Liu Z Chen Y Wang Y Li J . Decoding per- and polyfluoroalkyl substances (PFAS) in hepatocellular carcinoma: a multi-omics and computational toxicology approach. J Transl Med. (2025) 23:504. doi: 10.1186/s12967-025-06517-z 40317014 PMC12049027

[38] Boyd RI Shokry D Rennels BC Adam Y Powell C Johnson S . The role of lipid metabolism and peroxisome proliferator activation in mediating pro-cancer phenotypes of poly- and perfluoroalkyl substances in testicular cancer. Environ Toxicol Pharmacol. (2025) 120:104866. doi: 10.1016/j.etap.2025.104866 41237835 PMC13371115

[39] Soares LOS de Araujo GF Gomes TB Júnior SFS Cuprys AK Soares RM . Antioxidant system alterations and oxidative stress caused by polyfluoroalkyl substances (PFAS) in exposed biota: a review. Sci Total Environ. (2025) 977:179395. doi: doi: 10.1016/j.scitotenv.2025.179395 40245819

