# Supplementary material for: Per- and polyfluoroalkyl substances (PFAS) and cancer: knowledge mapping and emerging trends based on a bibliometric analysis
Source: Front Oncol. 2026 Oct 19;16:1880116. doi: 10.3389/fonc.2026.1880116 (PMC13724382; doi:10.3389/fonc.2026.1880116)
Supplement: Supplementary file 1 [file Table1.docx]

**Supplementary materials**

**Per- and Polyfluoroalkyl Substances (PFAS) and Cancer: Knowledge Mapping and Emerging Trends Based on a Bibliometric Analysis**

Siyin Liu^1, †^, Guoqing Zhang^2, †^, Meijing Wang^3^, Jiaguo Huang^4*^

^1^ Department of Breast and Thyroid Surgery, People's Hospital of Xinjiang Uygur Autonomous Region, Urumgi, Xinjiang, China.

^2^ Department of Clinical Nutrition, West China Hospital, Sichuan University, Chengdu, Sichuan, China.

^3^ College of Traditional Chinese Medicine, Xinjiang Medical University, Xinjiang, China.

^4^ Department of Urology, Affiliated Xiaoshan Hospital, Hangzhou Normal University, Hangzhou, Zhejiang, China.

† These authors contributed equally to this work

*** Correspondence:**

13735526508@163.com (Jiaguo Huang)

Table 1 Representative records excluded during relevance screening and reasons for exclusion

| No. | First author and year | Article title | Reason for exclusion |
| --- | --- | --- | --- |
| 1 | Zongxi Zhao  (2024) | Per- and poly-fluoroalkyl substances (PFAS) accelerate biological aging mediated by increased C-reactive protein | Examined biological aging and systemic inflammation rather than cancer |
| 2 | Rui Qu  (2024) | Per- and Polyfluoroalkyl Substances (PFAS) Affect Female Reproductive Health: Epidemiological Evidence and Underlying Mechanisms | Focused on female reproductive health outcomes rather than cancer |
| 3 | Anna S Young  (2021) | Per- and polyfluoroalkyl substances (PFAS) and total fluorine in fire station dust | Environmental exposure-monitoring study without cancer-related outcomes |
| 4 | Xiaoxi Yang  (2026) | Per- and polyfluoroalkyl substances and cardiovascular disease: A mechanistic and epidemiological synthesis | Focused on cardiovascular disease rather than cancer |
| 5 | Kajsa Ugelvig Petersen (2020) | Per- and polyfluoroalkyl substances and male reproductive health: a systematic review of the epidemiological evidence | Focused on male reproductive outcomes rather than cancer |
| 6 | Shiryn D Sukhram (2025) | PFAS Exposure, Mental Health, and Environmental Justice in the United States: Impacts on Marginalized Communities | Focused on mental health and environmental justice; no cancer-specific analysis |
| 7 | Gaurav Jha(2021) | Per- and Polyfluoroalkyl Substances (PFAS) in Integrated Crop-Livestock Systems: Environmental Exposure and Human Health Risks | Focused on PFAS contamination and exposure in agricultural systems; not specifically centered on cancer |
| 8 | Qi Qi  (2023) | Per- and polyfluoroalkyl substances activate UPR pathway, induce steatosis and fibrosis in liver cells | Investigated hepatotoxicity, steatosis, and fibrosis without a cancer model or cancer-specific endpoint |
| 9 | Qi Sun  (2018) | Plasma Concentrations of Perfluoroalkyl Substances and Risk of Type 2 Diabetes: A Prospective Investigation among US Women | Evaluated type 2 diabetes as the primary health outcome, without investigating cancer risk or progression. |
| 10 | Imari Walker-Franklin  (2025) | Preliminary Identification of PFAS and Other Emerging Contaminants in the French Broad River, NC Post-Hurricane Helene | Focused on environmental monitoring of PFAS and other contaminants; no cancer-related outcome was assessed. |
